# Time- and cost-effective production of untagged recombinant MVA by flow virometry and direct virus sorting

**DOI:** 10.1186/s12967-023-04353-7

**Published:** 2023-07-23

**Authors:** Daniela Boselli, Maddalena Panigada, Simona Di Terlizzi, Monica Romanò, Emanuele Canonico, Chiara Villa, Claudia Minici, Eelco van Anken, Elisa Soprana, Antonio G. Siccardi

**Affiliations:** 1 Division of Genetics and Cell Biology, San Raffaele Scientific Institute, Milan, Italy; 2 FRACTAL - San Raffaele Scientific Institute, Milan, Italy; 3 Università Vita-Salute San Raffaele, Milan, Italy

**Keywords:** Recombinant MVA, rMVA, Flow Virometry, Virus-Sorting, EEV and IMV virions, Hemagglutinin/egfp fusion protein, Red-to-Green Gene Swapping Method

## Abstract

**Background:**

Recombinant MVAs (rMVAs) are widely used both in basic and clinical research. Our previously developed Red-to-Green Gene Swapping Method (RGGSM), a cytometry-based Cell-Sorting protocol, revolves around the transient expression of a green fluorescent cytoplasmic marker, to subsequently obtain purified untagged rMVA upon loss of that marker by site-specific recombination. The standard RGSSM is quite costly in terms of bench work, reagents, and Sorting Facility fees. Although faster than other methods to obtain recombinant MVAs, the standard RGSSM still is time-consuming, taking at least 25 days to yield the final product.

**Methods:**

The direct sorting of fluorescent virions is made amenable by the marker HAG, a flu hemagglutinin/EGFP fusion protein, integrated into the external envelope of extracellular enveloped virions (EEVs). Fluorescent EEVs-containing supernatants of infected cultures are used instead of purified virus. Direct Virus-Sorting was performed on BD FACSAria Fusion cell sorter equipped with 4 lasers and a 100-mm nozzle, with 20 psi pressure and a minimal flow rate, validated using Megamix beads.

**Results:**

Upon infection of cells with recombinant EEVs, at the first sorting step virions that contain HAG are harvested and cloned, while the second sorting step yields EEVs that have lost HAG, allowing to clone untagged rMVA. Because only virion-containing supernatants are used, no virus purification steps and fewer sortings are necessary. Therefore, the final untagged rMVA product can be obtained in a mere 8 days.

**Conclusions:**

Altogether, we report that the original RGSSM has been markedly improved in terms of time- and cost efficiency by substituting Cell-Sorting with direct Virus-Sorting from the supernatants of infected cells. The improved virometry-based RGGSM may find wide applicability, considering that rMVAs hold great promise to serve as personalized vaccines for therapeutic intervention against cancer and various types of infectious diseases.

**Graphical Abstract:**

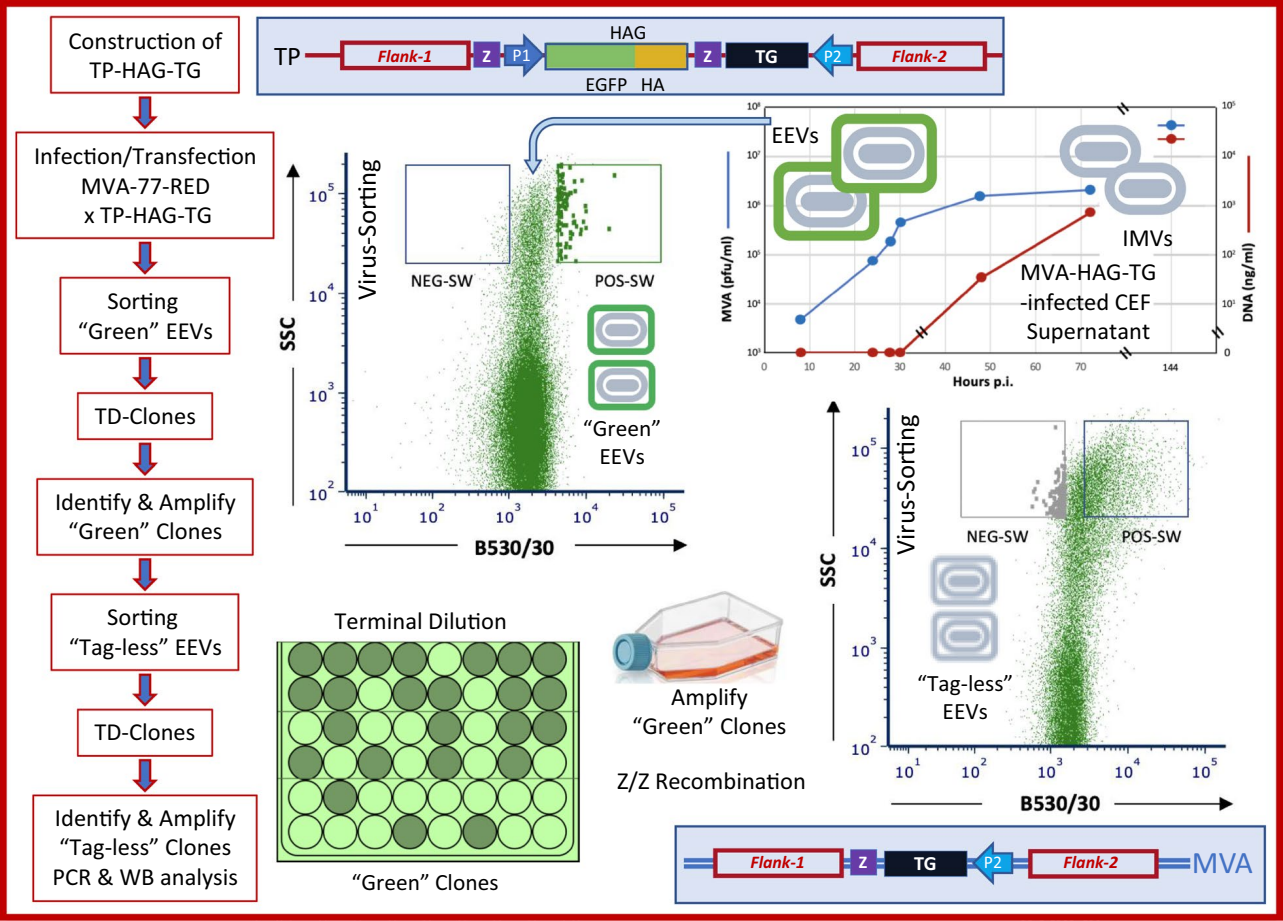

## Introduction

Modified Vaccinia virus Ankara (MVA) was derived from the Chorioallantois Vaccinia virus Ankara strain by Anton Mayr in Munich in the 1970s, as an attenuated alternative to the conventional smallpox vaccine [1]. In the early 1990s, MVA was repurposed to be used as a vaccine vector [2], and since then, various recombinant MVAs (rMVAs), coding several different antigens of therapeutic value or scientific interest have been produced to serve in basic and/or clinical research [3, 4]. The interest in rMVA for therapeutic purposes is indeed growing still. For instance, rMVA has been proposed to serve as a vector for personalized tumor vaccines [5].

Unfortunately, currently available protocols for rMVA production are tedious and costly. Hence, better streamlined protocols are urgently needed for rMVA production to be readily translated in clinical treatments. We have developed the Red-to-Green-Gene-Swapping-Method (RGGSM) to construct rMVAs already more than a decade ago [6, 7]. In brief, our fluorescence-based protocol, which allows to select rMVA-producing cells that fluoresce in green over parental MVA-producing cells that fluoresce in red, requires iterative Flow Cytometry sorting steps. The swap in fluorescent marker occurs as a result of homologous recombination because the cassette, containing both EGFP and the transgene of choice, is flanked by stretches of Vaccinia DNA [8], identical to those flanking the HcRed 1.1 red fluorescent reporter gene in parental MVA. Once the red parental MVA-producing cells have been counter-selected for, we sort from the pool of cells that produce “green” rMVA those that, in turn, have lost EGFP. The elimination of the tag is critical for the production of rMVAs that might serve as experimental vaccines; to this purpose, the deletion of EGFP is obtained through recombination due to EGFP being flanked by so-called Z-repeats (283-bp sequences from *E.coli* LacZ gene) on either side [8].

Following this protocol, from colorless infected cells untagged rMVAs are obtained, that are suitable for both research [9–14] and biotechnological purposes [5, 15]. The RGSM offers an elegant method to produce untagged rMVA, nevertheless the procedure is laborious, time-consuming, and costly. Moreover, virus is routinely purified after each Cell-Sorting step, in order to keep well-characterized back-up intermediates.

Over the past decade, Flow Virometry [16–19] is in ascendency. Thus far, more than a dozen different viruses ranging in size from 40 nm to giant have subjected successfully to Flow Virometry [20]. Importantly, also vaccinia viruses, which are brick-shaped and up to 350 nm in size [21] have been shown to be amenable to analysis and sorting [22, 23]. Given these insights, we set out to exploit these technical advances to improve our RGGSM. We report here that by choosing virometry-based sorting (Virus-Sorting) over cytometry-based (Cell-Sorting) steps, the resulting ameliorated protocol faithfully yields cloned rMVA in shorter time and more effortlessly. Moreover, the costs of reagents for cell cultures, virus purification, and sorting facility fees are sensibly cut down.

## Materials and methods

### Construction of transgene transfer plasmids (TP)

**Fig. 1:**
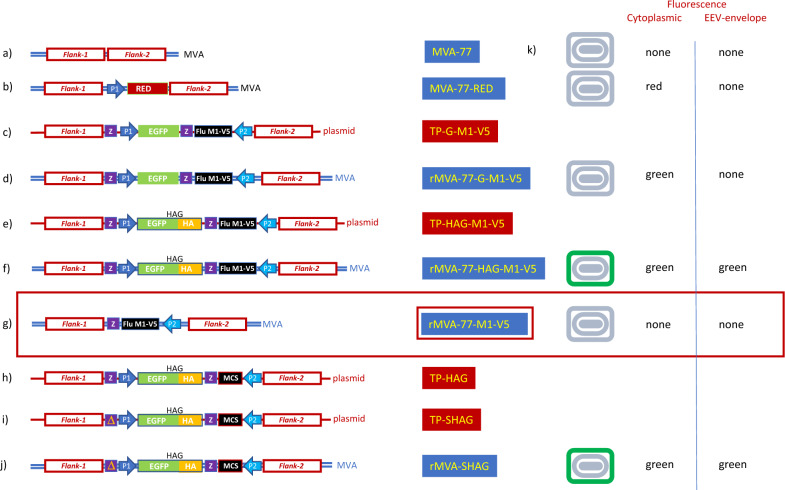
Schematic representation of marker and transgene cassettes of Transfer Plasmids and rMVAs. **a** and **b** MVA-77 is a TD-clone from a Bavarian Health Department vaccine sample lyophilized in 1977. Derivative MVA-77-RED is the acceptor for all infection/transfection-driven recombinations within the Flank-1 and Flank-2 sequences. **c** and **d** TP-G- M1-V5 and rMVA-77-G- M1-V5. Transfer Plasmid and the derivate rMVA carrying EGFP as fluorescent tag (under the control of the synthetic promoter sP (P1) and, as transgene, the Flu CA/09 membrane protein M1 gene, tagged with epitope V5, inserted in the multiple cloning site (MCS) and controlled by P7.5 early/late promoter (P2). **e** and **f**. TP-HAG- M1-V5 and rMVA-77-HAG- M1-V5. Transfer Plasmid and the derivate rMVA carrying HAG as fluorescent tag and M1-V5 as transgene. **g** Final untagged rMVA-77- M1-V5 product. **h**, **i** and **j** TP-HAG, TP-SHAG and rMVA-SHAG. Starting from TP-HAG, the upstream Z repeat was deleted, as marked by “∆” in the yellow box (TP-SHAG), allowing SHAG to serve as a “stable green” marker. **k** Expected fluorescent phenotypes of rMVA-infected cells (cytoplasmic) and of the virions themselves (EEV-envelope)

The flu HA/EGFP fusion protein (HAG) was constructed by inserting the HA gene from Flu CA/09 virus at the 3’ end of the EGFP gene in plasmid BlueScript SK(−)sP-EGFP. Within the already described Transfer Plasmid Green TP-G [7], the EGFP gene was substituted by the HAG fusion protein yielding Transfer Plasmid TP-HAG. TP-SHAG was then derived from TP-HAG by deleting a region containing one of the two Z-repeats, necessary to excise the EGFP gene by homologous recombination [8] to yield the final untagged rMVA (Fig. 1).

As a transgene, we opted for the Flu CA/09 membrane protein M1 gene, tagged with epitope V5 [24]. It was inserted in the MCS of both TPs, yielding TP-G- M1-V5 and TP-HAG- M1-V5. The same transgene, in a previous construct (here called rMVA-M1-V5-Rome) was tested as an experimental vaccine [12]. In this paper, rMVA-M1-V5-Rome DNA is used as a positive control for PCR and Western Blot analyses. Expression of the EGFP and HAG marker genes, and M1-V5 is driven by vaccinia synthetic promoter sP [25], and, respectively, the P7.5 early/late promoter [26].

### Recombination assays to obtain rMVAs

Fresh Chick Embryo Fibroblasts (CEFs) were prepared from 11-day old embryonated Specific Pathogen Free chicken eggs (Charles River, Paris, France) and maintained in a serum-free medium containing 10 ng/mL EGF (VP-SFM, GIBCO), supplemented with 2% glutamine and 1% Pen/Strep, as previously described [6, 7]. MVA-77 is a TD-clone from a Bavarian Health Department vaccine sample lyophilized in 1977 (lot # 77–45, 1977), which we obtained through courtesy of Professor Volker Erfle, Institute of Molecular Virology, TU, Munich, Germany.

The MVA-77-RED derivative was constructed by the insertion of the HcRed 1.1 gene between the so-called Flank-1 and Flank-2 sequences [8] of MVA-77 (Fig. 1, lines a and b), identically as described for the "MVA-RED" acceptor virus that we used in previous work [7]. MVA-77-RED is used here as the acceptor for all infection/transfection-driven recombination, facilitated by the Flank-1 and Flank-2 sequences. The resulting swap of the HcRed 1.1 gene with the cassettes present in the TP vectors, yielded the following viruses: rMVA-77-HAG-M1-V5, from recombination with TP-HAG-M1-V5 (Fig. 1 lines e and f), rMVA-77-SHAG, from recombination with TP-SHAG (Fig. 1, lines i and j), and rMVA-77-G-M1-V.5, from recombination with TP-G-M1-V5 (Fig. 1, lines c and d). Pure rMVA-77-SHAG and rMVA-77-G-M1-V.5 were obtained through our standard cytometry-based RGGSM [7].

### Transgene expression analysis by immunoblotting

CEF cells (ca. 1 × 10^6^), infected with rMVA at m.o.i. 5 (or mock-infected) were lysed with sample buffer (62.5 mM Tris–HCl, pH 6.8, 2% SDS, 100 mM dithiothreitol, 10% glycerol, 0.1 mg/mL bromophenol blue), and immunoblotting was performed as previously described [9]. In brief, protein samples were resolved by 10% SDS–PAGE and transferred to nitrocellulose. Reversible Ponceau staining was used as a loading control [27]. The HAG fusion protein was detected using chicken anti-H1N1 polyclonal antibodies (Istituto Zooprofilattico Sperimentale Tre Venezie, Padova, Italy), and the M1-V5 transgene was detected using the anti-V5 monoclonal antibody V5-10 (Sigma–Aldrich), respectively followed by HRP-conjugated rabbit anti-chicken Ig, or anti-mouse Ig antibodies (DakoCytomation, Denmark A/S). Western blots were developed by enhanced chemiluminescence (Amersham ECL; GE Healthcare, UK) autoradiography.

### Cytometry-based RGGSM

The cytometry-based RGGSM was performed essentially as described [7]. For the first round of selection, CEFs were collected, by trypsinization, upon 48 h infection with MVA-77-RED and transfection with the TP constructs. Cells were then washed and kept on ice until Cell-Sorting on BD FACSAria Fusion (BD Bioscience) equipped with four lasers: Blue (488 nm), Yellow/Green (561 nm), Red (640 nm) and Violet (405 nm). R670/30 (red) and B530/30 (green) channels were chosen to sort green from red fluorescent or non-fluorescent cells in the Positive Sorting Window (POS-SW). The collected cells were lysed, and lysates were subjected to freeze, thaw, and sonication (F/T/S).

Lysates containing infectious virus particles were then diluted 1:100 and used to infect a fresh CEF culture in the presence of 1 μM Cytochalasin D (Sigma-Aldrich), which reduces superinfection as it is an inhibitor of extracellular virion release. Cells were then harvested 24 h post infection (*p.i.*), for another round of Cell-Sorting as described above, followed by TD. Upon amplification of the TD cultures, the procedure was repeated for a third round of Cell-Sorting. This time, the target selected cells were no longer green fluorescent and were selected in the Negative Sorting Window (NEG-SW). Upon TD of the selected cells, clones were selected for further analysis.

### Transgene analysis by PCR

The presence of the transgene M1 was validated by M1-specific PCR, using internal primers (Forward primer: 5ʹ-GGGCCCATGAGCCTGCTGACCGAGG-3ʹ; Reverse primer: 5ʹ-AGGCGCGCCTTAGTCCAGGCCCAGCAGG-3ʹ), and using a Taq DNA Polymerase recombinant (Invitrogen cat #10342–020) in an Mastercycler pro Thermal Cycler (Eppendorf) followed by agarose gel electrophoresis.

### Confocal and fluorescence microscopy

For fluorescent microscopy, CEFs infected by either rMVA-G- M1-V5 or rMVA-77-SHAG were grown on coverslips and fixed by 4% paraformaldehyde, permeabilized, saturated and processed for immunofluorescence. Phalloidin–Tetramethylrhodamine B isothiocyanate (TRITC; Sigma-Aldrich cat. N P1951) was used to identify filamentous actin. Fluorescent images were acquired using GE healthcare DeltaVision Ultra microscope equipped with a 60X oil-immersion lens (ALEMBIC, Milan, Italy). Images were then processed with ImageJ and Adobe Photoshop.

For live fluorescence microscopy of virus-infected CEF cultures displaying red fluorescence due to expressing HcRed1.1, and/or green fluorescence due to expressing EGFP or HAG were visualized using an inverted Olympus IMT-2 microscope (Olympus Optical Co., Milan, Italy). Images were taken by a digital camera (Olympus CAMERA C2000).

### Infectivity and cell lysis assays

Following infection and transfection of CEFs, as described above for the cytometry-based RGGSM, supernatants were collected at various time points *p.i.*, and infectivity of supernatants (*i.e.* the viral titer) was determined by focus assays (by live fluorescent microscopy). The contamination of cellular materials in the supernatants was measured by Quant-iT™ PicoGreen^®^ dsDNA Assay Kit (ThermoFischer-Molecular Probes, Inc.) according to the manufacturer’s instructions. Pico Green is a nucleic acids-binder permeant dye, used as a sensitive index of cell lysis [28].

### Virus-Sorting

Following infection and transfection, as described above for the cytometry-based RGGSM, supernatants were collected at 30 h *p.i.*, and kept on ice before Virus-Sorting. For EEVs, high resolution flow analysis was performed with a BC CytoFLEX S (Beckman Coulter) equipped with four lasers: Blue (488 nm), Yellow/Green (561 nm), Red (638 nm) and Violet (405 nm) and with a second side scatter on the violet laser (Violet SSC). A threshold of 1000 channels on Violet SSC was used to remove part of background signal. Amplifier settings for forward scatter (FSC) and Violet SSC as well as for any fluorescence channel were set in logarithmic mode.

Scatter channel calibration was obtained by using Biocytex Megamix beads, a mix of fluorescent beads (of 100, 300, 500, and 900 nm diameter) which emit in the B525/40 channel of the BC CytoFLEX S. Conversely, Virus-Sorting was performed on BD FACSAria Fusion equipped with four lasers: Blue (488 nm), Yellow/Green (561 nm), Red (640 nm) and Violet (405 nm). The threshold was set on the B 530/30 channel. The 100 μm nozzle was used, and sheath fluid pressure was set at 20 psi. The event rate was set at < 5000 events/second. For bulk recovery, we used the 4-way-purity mode, collecting several thousand events from Positive or Negative Sorting Windows (POS-SW & NEG-SW). A re-analysis of sorted samples was performed in order to confirm sorting efficiency. For SYTO 13 staining an aliquot of the EEV suspension was diluted 1:10 in PBS or in a 200 nM solution of SYTO 13 (Molecular Probes) [29].

### Data analysis

All raw data obtained with the two different instruments (BC CytoFLEX and BD FACSAria Fusion) have been processed with a third-party software, FCS Express 7 (De Novo software).

### Terminal dilution

For cytometry-based RGGSM, sorting in bulk green fluorescent or untagged rMVA-infected cells yielded samples containing several thousand entities which were terminally diluted on CEF monolayers (seeded the night before), as previously described [7].

Instead, for virometry-based RGGSM, green fluorescent or untagged EEVs were sorted in bulk (a few thousand events, as counted by the sorting instrument), treated by F/T/S, subjected to several 1:3 dilutions, and seeded on CEF monolayers in Microtiter plates (48 replicas/dilution). To obtain veritable clones, a dilution was considered suitable only if at least 35/48 microcultures were uninfected, which, for the Poisson distribution, corresponds to 0.7 focus-forming-units (ffu)/culture (with a 7:1 ratio of single to multiple infections).

Marker-free poxvirus vectors have been designed to comply with the regulatory guidance from both the US Food and Drug Administration and European Medicines Agency.

## Results

### Validation of a membrane-bound green reporter for RGGSM

For the original RGGSM we used cytoplasmic expression of EGFP as a marker of successful MVA recombination [6, 7]. Yet, little if any cytoplasmic EGFP gets to be incorporated into rMVA virions, precluding them to be selectable through Virus-Sorting based on green fluorescence. Thus, as a first step to adapt RGGSM to exploit Flow Virometry (and, eventually, Virus-Sorting), we decided to substitute the cytoplasmic EGFP with a membrane-bound EGFP fusion protein to mark rMVA external membranes.

Rather than employing already published EGFP-EEV membrane fusion proteins [30–32], we produced our own membrane marker protein, by tagging flu hemagglutinin (HA) with EGFP. The choice of HA derives from previous work [7], where we used antibody-labelled transgenic HA on the surface of infected cells to analyze the recombination kinetics leading to the loss of red and green markers. Since transgenic vaccinia and MVA viruses display HA on the cell membrane of infected cells [7, 33], and since plasma membrane proteins co-purify with EEV, but not with IMV particles, when analyzed by immunoblotting [33], we reasoned that our membrane marker HAG would be displayed on EEV particles and could be used as an EEV-fluorescent tag.

We constructed the HAG reporter gene by using EGFP as an N-terminal fusion to HA from influenza CA/09, with Z-repeats flanking HAG on either side (Z-HAG-Z) to allow its excision later (Fig. 1, line h). Firstly, we validated the effectiveness of HAG as a transient marker for insertion of transgenes into MVA by obtaining a rMVA expressing the influenza CA/09 membrane protein M1 gene, tagged with epitope V5 (M1-V5) [24] with our standard Cell-Sorting RGGSM, also to obtain a reference to whom the Virus-Sorting RGGSM results can be compared. The cassette containing the Z-HAG-Z/ M1-V5 tandem was placed into our standard Transfer Plasmid (TP) and thereby inserted between the vaccinia-derived sequences Flank-1, and Flank-2, on either side to sustain homologous recombination with MVA-77-RED, as described previously [6].

**Fig. 2:**
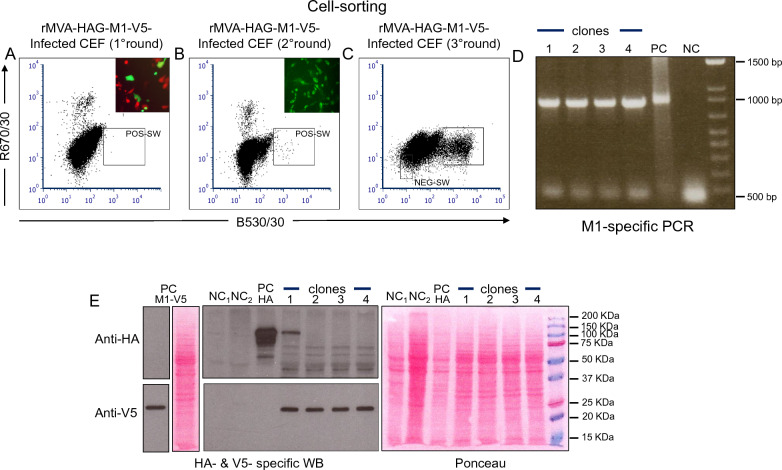
rMVA- M1-V5 production by cytometry-based RGGSM. **A** infection/transfection of CEF cells with MVA-77-RED and TP-HAG- M1-V5 DNA. Green fluorescent cells were sorted from the POS-SW, yielding a mixed “green + red” cell population (inset). **B** An enriched green fluorescent population (inset) were obtained by a second round of Cell-Sorting, from which, after F/T/S, green virus clones were isolated by TD. In this experiment, approximately 5000 events were sorted and 8/48 “green” microcultures were obtained at TD, which corresponds to 0.19 ffu/culture, with a 13:1 ratio of single to multiple infections. **C** Flow cytometry of CEFs infected with virus isolated from a green clone. Untagged rMVA-infected cells were sorted from a double-negative NEG-SW. Finally, after F/T/S, untagged virus clones were isolated by TD. In this experiment, approximately 5000 events were sorted and 6/48 "green" microculture were obtained at TD, which corresponds to 0.12 ffu/culture, with a 16:1 ratio of single to multiple infections. **D** M1-specific PCR confirmed the presence of the M1 transgene in both green HAG-tagged (lane 1) and untagged clones (lanes 2 to 4) as in the TP-HAG- M1-V5 construct, which served as a positive control (PC), but unlike in a sample from MVA-77-RED-infected CEFs, which served as a negative control (NC). **E** HA-specific WB confirmed the presence of the HAG fusion gene in the control green isolate (lane 1) and its absence in untagged clones (lanes 2 to 4). CEFs infected with rMVA-SHAG (see Methods) served as a positive control (PC HA). The V5-specific WB confirms the presence of the M1-V5 protein in all isolates (lanes 1 to 4). CEFs infected with rMVA-Rome- M1-V5 (see Methods) served as a positive control for the presence of the M1-V5 transgene (PC M1-V5). Non-infected CEFs and MVA-77-RED-infected CEFs served as negative controls (NC1 and NC2, respectively)

CEF cells were then transfected with DNA of the resulting construct, TP-HAG- M1-V5 (Fig. 1, line e) and infected with MVA-77-RED (Fig. 1, line b) to obtain rMVA-77-HAG- M1-V5 (Fig. 1, line f). These infected/transfected cells, 24 h later, were submitted to the standard Cell-Sorting RGGSM. As expected, a first Cell-Sorting step revealed an enrichment of green cells, as determined by FACS analysis (Fig. 2A), and by live fluorescence microscopy (Fig. 2A, inset). Because red foci were still more frequent than green foci, a second round of infection of CEFs with the virus harvested from the sorted cells was needed, followed by a subsequent round of Cell-Sorting resulting in a large increase in the number of green events (Fig. 2B and 2B inset).

Clonality of the sorted cells was obtained at the appropriate terminal dilution (TD; see Methods) and among the clonal “green” microcultures one was chosen for amplification and a further round of Cell-Sorting. At this point, the disappearance of the “red” population of events was almost complete, while the “green” population of events was prevalent (Fig. 2C). Cell-Sorting from a double negative window (NEG-SW), followed by F/T/S and TD, finally yielded candidate untagged rMVA clones (Fig. 1, line g).

We selected three of these clones, together with one "green" clone for comparison, for further characterization. PCR using M1-specific primers confirmed that all four clones harbored a full-length M1 copy (Fig. 2D), which was also corroborated by immunoblotting, as the clones were positive for the V5 epitope (Fig. 2E). All except the "green" control clone were negative for staining with anti-HA antibodies (Fig. 2E), indicating that all three candidate clones had lost HAG, and thus represented *bona fide* clones of untagged M1-V5 rMVA.

Altogether, we concluded that the standard RGGSM was successful also upon replacing cytoplasmic EGFP with membrane-bound HAG as a transient marker for recombination. The whole procedure, including three rounds of virus purification and Cell-Sorting, took about 25 days, as is the routine (Fig. 8A).

### rMVA can be harvested even before cell lysis

Next, we set out to establish conditions for fluorescent sorting of rMVA EEVs. To this end, we first created a "stable green" HAG construct (SHAG) by removal, in the TP, of one of the Z repeat sequences flanking HAG (Fig. 1 line i). After recombination with MVA-77-RED, TP-SHAG thus yielded rMVA-77-SHAG (Fig. 1 line j), excluding the potentially confounding effect of the HAG marker getting (prematurely) lost due to Z-repeats driven recombination.

**Fig. 3:**
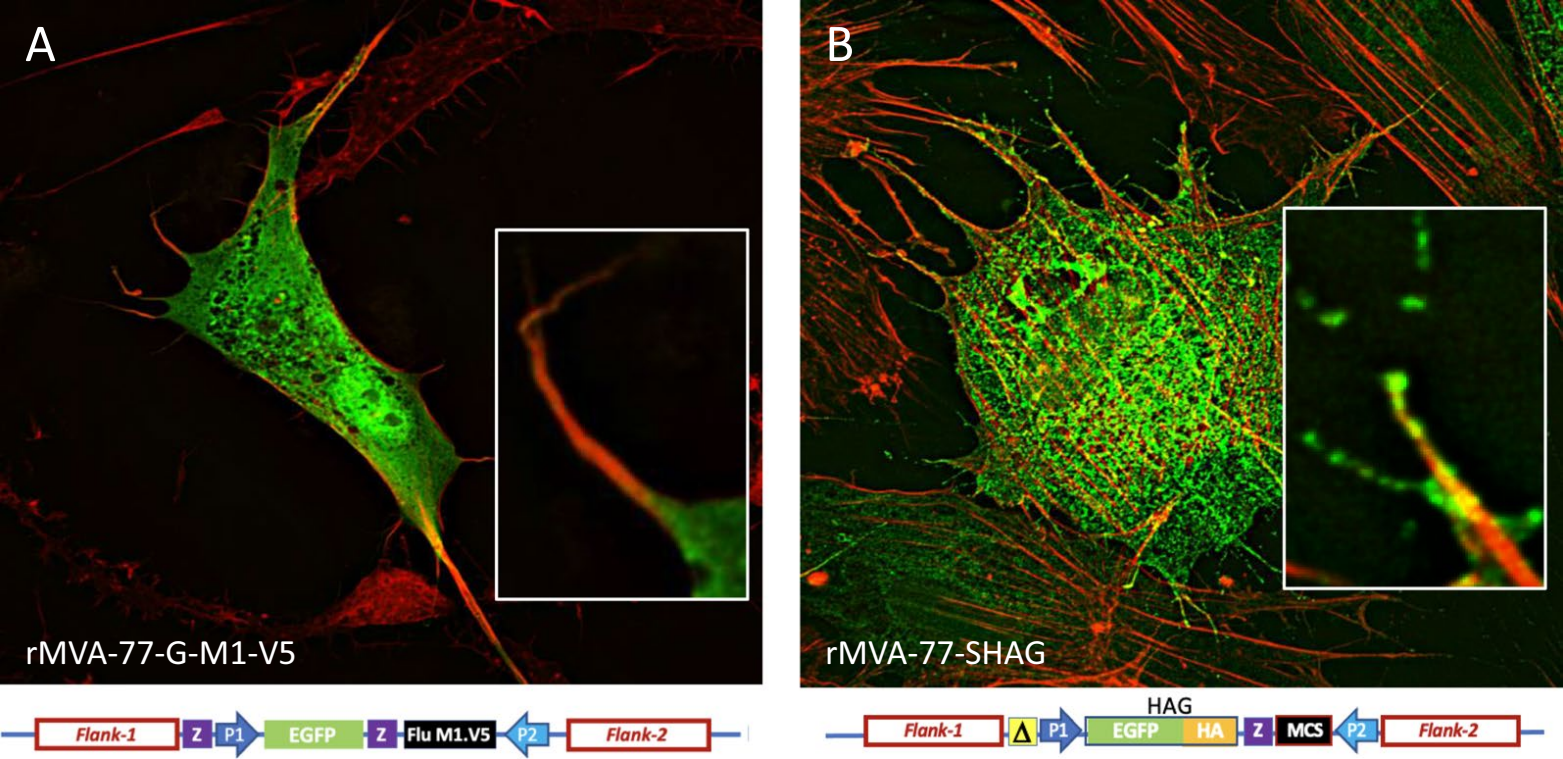
A membrane bound marker for RGGSM is expressed at the cell surface. **A** EGFP-expressing rMVA-77-G-M1-V5-infected CEFs show a “cytoplasmic green” signal **B** rMVA-77-SHAG-infected CEFs show a "stable green" signal displayed also at the cell surface and on actin projections mainly as membrane vesicles. Actin was labeled in red by phalloidin-TRITC. Transgene cassettes schemes below the micrographs are detailed in Fig. 1

We then infected CEFs with either "cytoplasmic green" rMVA-77-G-M1-V5 (Fig. 1 line f) or “stable green” rMVA-77-SHAG and analyzed cells by fluorescence microscopy. As expected, in rMVA-77-G-M1-V5-infected CEFs the green fluorescence is diffused in the cytoplasm but absent from the cell surface and from actin projections (labelled in red by phalloidin-TRITC) (Fig. 3A). Conversely, both structures fluoresce in green in rMVA-77-SHAG-infected CEFs (Fig. 3B), indicating that HAG indeed is displayed on the cell surface and on actin projections, mainly in the form of membrane vesicles. Notably, it is unlikely to see EEVs in this assay, because EEVs are secreted in the supernatant and, typically, get to be lost during washing procedures.

**Fig. 4:**
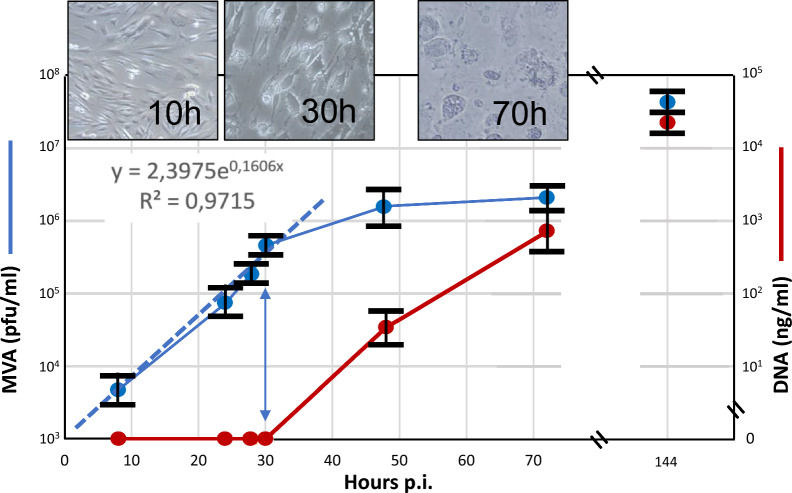
Substantial release of virions in supernatants of MVA-infected cells. Supernatants from rMVA-77-SHAG- infected CEFs were harvested at various time points upon infection. Viral titers (ffu/ml, blue curve) and lysis-released cellular dsDNA (ng/ml, red curve) were determined; titer and DNA values are averages ± SD of 3 replicas. Excel log trend and equation for the first part of the viral titer curve are shown. Photographic inserts show cell morphology at 10, 30 and 70 h *p.i*

Next, we harvested supernatants of rMVA-77-SHAG-infected CEFs at various time points upon infection and found that during the first 30 h post-infection (*p.i*.) there was an exponential increase in infectivity of supernatants up to 5 × 10^5^ ffu/ml (Fig. 4, blue graph), confirming a substantial release of EEVs from rMVA-77-SHAG-infected CEFs. Yet, during this time window, cell lysis was negligible, since there was neither an increase in release of cellular dsDNA in the supernatant (Fig. 4, red graph), nor any visible sign of cell degeneration by microscopy (Fig. 4, 10 and 30 h insets).

From 30 h *p.i.* onwards, however, dsDNA began to accumulate in supernatants, reflecting the onset of cell lysis. In fact, at 70 h *p.i.* cell degeneration was also microscopically manifest (Fig. 4, 70 h inset). Cell lysis eventually led to a further 100-fold increase in infectivity at 144 h *p.i.*, as most of the remaining intracellular mature viruses (IMVs) were liberated from the cells. Yet, we reasoned that upon the onset of cell lysis, cell debris and immature viral particles inevitably would contaminate supernatants to such extent to obfuscate the rMVA EEV particles we wished to select by virus sorting. Hence, we concluded that we should focus instead on those rMVA EEVs that are shed from the cells in substantial amounts already prior to cell lysis.

### rMVA can be detected by Flow Virometry

As a second step to establish conditions for fluorescent sorting of rMVA EEVs, we exploited Megamix beads to set the instruments, as they emit green fluorescence and cover a size range of 100–900 nm, which encompasses the 250–350 nm size range of EEVs. To set scatter channels suitable for virus detection, we analyzed Megamix beads at low flow rate on a BC CytoFLEX S instrument.

**Fig. 5:**
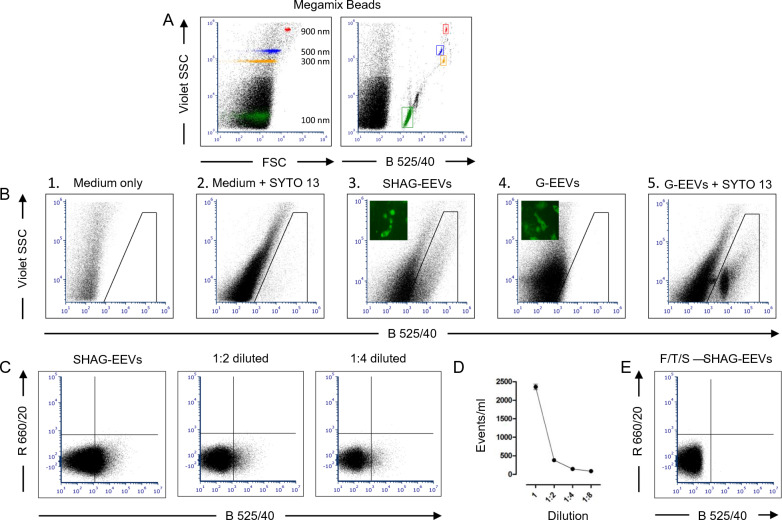
Visualization of fluorescent EEVs by Flow Virometry analysis. **A** Physical, FSC vs. Violet SSC plot (left panel), and fluorescence, B525/40 (green) vs. Violet SSC plot (right panel), characterisation of Megamix beads (900 nm—red, 500 nm—blue, 300 nm—yellow and 100 nm—green dots) by BC CytoFLEX S analysis, setting the instrument for flow virometry. **B** CEFs were infected with either rMVA-77-SHAG or rMVA-77-G- M1-V5, and supernatants were harvested 30 h *p.i.* Supernatants were analysed with the settings established using the Megamix beads (B525/40 vs. Violet SSC), revealing signals for entities with the “stable green” surface marker (SHAG-EEVs, left panel), but not so for the “cytoplasmic green” marker (G-EEVs, middle panel), unless these entities were stained in the DNA with the SYTO 13 dye (G-EEVs + SYTO 13, right panel). **C** and **D** BC CytoFLEX S analysis with B 525/40 (green) versus R 660/20 (red) settings of SHAG-EEVs. The green fluorescence is progressively lost with sample dilution. E) F/T/S treatment of SHAG EEVs abolishes the green signal, confirming its membrane localization

In forward scatter (FSC) versus side scatter (SSC) plots, using the 405 nm (violet) laser for SSC, 100 nm beads, marked by green dots (Fig. 5A, left panel) were indistinguishable from noise, while signals were already evident for beads with a diameter of 300 nm (yellow dots), or larger, such as the 500 nm (blue dots), and 900 nm beads (red dots) (Fig. 5A, left panel). However, all the four kinds of beads became distinguishable once we exploited the green fluorescence for their detection using the B 525/40 channel (Fig. 5A, right panel).

We then analyzed supernatants from CEFs that were infected for 30 h with either "stable green" rMVA-77-SHAG, or "cytoplasmic green" rMVA-77-G- M1-V5 (Fig. 5B, insets) on the same instrument, with the previously established settings. Excitingly, in the window of interest, where we expected to detect EEVs, a diffuse cloud of green fluorescent entities emerged for the “stable green” rMVA as they display HAG at their surface (SHAG-EEVs, Fig. 5B, left panel), but not for the “cytoplasmic green” rMVA (G-EEVs, Fig. 5B, middle panel), evidently for lack of incorporation of EGFP into the viral particles. Indeed, staining with the green fluorescent DNA-binding dye SYTO 13 [29] readily made also the G-EEVs visible in the B 525/40 channel (Fig. 5B, right panel).

The signal for SYTO 13-stained G-EEVs (on DNA) showed a uniform distribution, suggesting the presence of non-aggregated single virions. The signal for SHAG-EEVs (on membranes), in contrast, showed a more diffuse distribution, reflecting a greater heterogeneity in dimensions and suggesting that other membrane vesicles, such as those seen in Fig. 3B, are detected as EGFP-positive events. Indeed, F/T/S of sorted SHAG-EEVs, abrogated the green fluorescence (Fig. 5C), in line with the notion that HAG-containing membranes were disrupted. We reckoned that the resulting disentangled virions (essentially, IMVs) were suitable for TD purposes. Since we analyzed the samples in “red” (R 660/20) versus “green” (B 525/40) plots, we could moreover confirm that SHAG-EEVs displayed no significant readout in the "red" channel (Fig. 5C).

### Flow Virometry-based sorting of rMVA

**Fig. 6:**
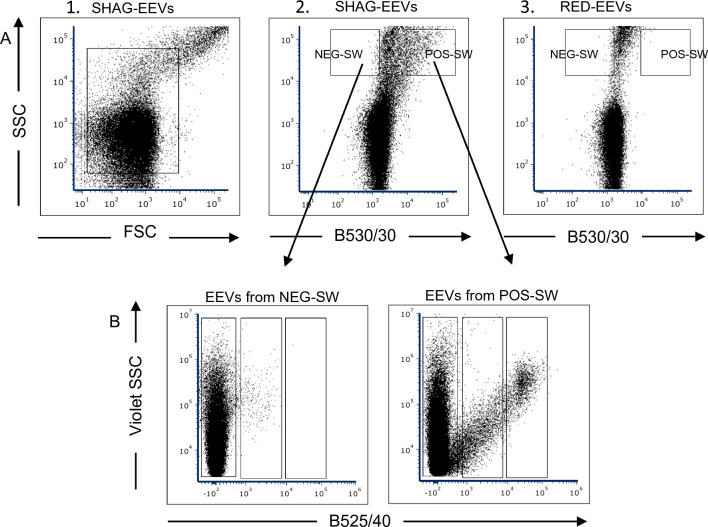
Flow Virometry sorting of green fluorescent rMVA virions. **A** Supernatants at 30 h *p.i.* of rMVA-77-SHAG- or rMVA-77-RED- infected CEFs (SHAG-EEVs and RED-EEVs) were first analysed on BD FACSAria Fusion, based on their physical characteristics (FSC vs. SSC plot, left panel) and a sorting window (rectangle) was chosen such as to exclude larger particles including cells. Next, entities selected from that sorting window were sorted based on green fluorescence (B530/30 vs. SSC plot, middle panel). NEG-SW and POS-SW were then set for further analysis. The POS-SW was defined such that there were no RED-EEVs detectable for that window (right panel). **B** The two sorted populations from the NEG-SW and POS-SW were re-analyzed by BC CytoFLEX S based on physical characteristics (Violet-SSC) and green fluorescence (B525/40), confirming the enrichment of green fluorescent entities (reflecting the presence of SHAG-EEVs) from the POS-SW, as compared to those from the NEG-SW

Having verified that SHAG-EEVs can be detected by Flow Virometry on an analytical instrument (BC CytoFLEX S), we performed the actual Virus-Sorting of SHAG-EEVs on the Cell Sorter BD FACSAria Fusion. From the supernatants of SHAG-EEV-producing CEFs, we first selected entities that based on FSC/SSC encompassed the EEVs but not the cells (Fig. 6A, left panel). Then, this selection was sorted based on green fluorescence, which allowed to define NEG-SW and POS-SW suitable to discriminate between HAG-negative and HAG-positive populations of events (Fig. 6A, middle panel). With such settings, hardly any signal was present in the POS-SW using samples from supernatants of MVA-77-RED-infected CEFs (Fig. 6A, right panel), confirming that the POS-SW faithfully selects green fluorescent events (among which HAG-positive EEVs).

The two sorted populations (respectively from NEG-SW, and from POS-SW) were then analyzed by BC CytoFLEX S, which further confirmed a significant enrichment of green fluorescent events derived from the POS-SW in comparison to the NEG-SW (Fig. 6B). At the same time these findings demonstrate that the NEG-SW effectively filters out green virions. Seeded on CEF monolayers, isolated green foci (*e.g.*, 150 ffu/5000 sorted events) confirmed that at least a fraction of the sorted green entities were indeed "green" virions. The large discrepancy between "focus forming units" and sorted events is in line with the notion that only 1 out of 30 virion particles is a viable focus forming unit [34] and that other non-virionic membrane-bound vesicles are sorted together with the virions (data not shown).

### Flow Virometry-based RGSSM to produce untagged rMVA

Having established that green fluorescent EEVs can be sorted from supernatants of rMVA-infected cells, and that these EEVs can be isolated as individual virions, we finally tested the usefulness of this approach for Virus-Sorting of rMVAs. Since RGSSM revolves around selecting rMVAs that are “transiently green”, as to obtain untagged rMVA, we employed again the “transiently green” rMVA-77-HAG- M1-V5-producing CEFs. At 30 h *p.i.,* we sorted from the supernatants green fluorescent entities, which appeared in the POS-SW, as we had defined above for the SHAG-EEVs (Fig. 6A, middle panel) to obtain “transiently green” HAG-EEVs. The sorted sample was subjected to F/T/S and terminally diluted into CEF microcultures, yielding pure green fluorescent clones (see Methods).

**Fig. 7:**
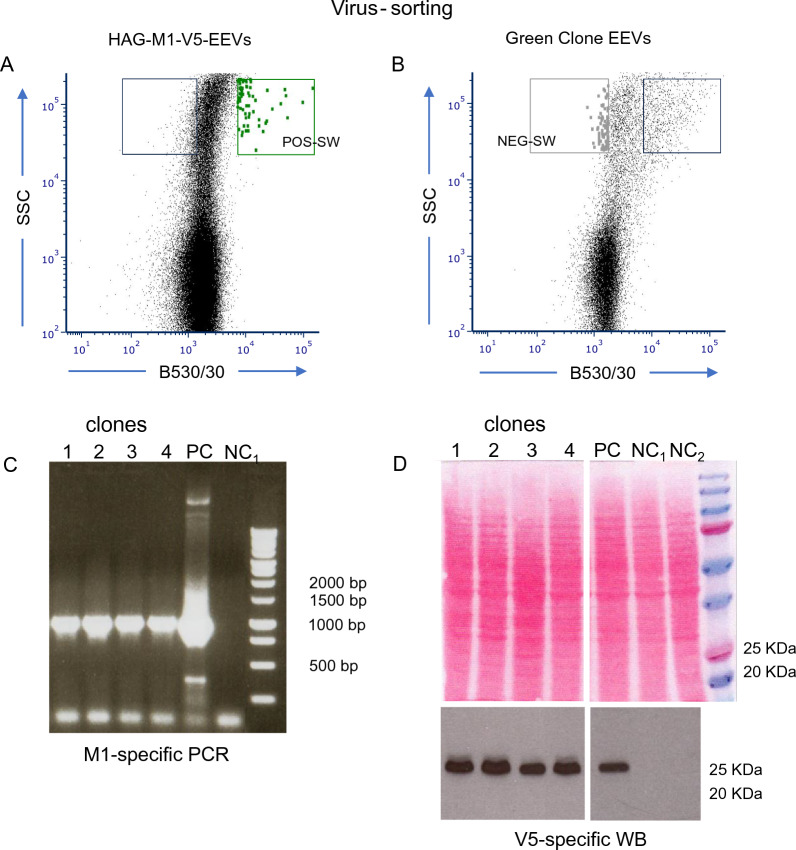
Untagged rMVA-M1-V5 production by virometry-based RGGSM. **A** Infection/transfection of CEF cells with MVA-77-RED and TP-HAG- M1-V5 DNA. Supernatants at 30 h were directly subjected to Virus-Sorting (BD FACSAria Fusion) based on green fluorescence and physical characteristics (B530/30 vs. SSC). Candidate rMVAs, HAG-M1-V5-EEVs were sorted in bulk from the POS-SW as defined in Fig. 6A, F/T/S-treated and subjected to TD. In this experiment, approximately 3500 events were sorted and 10/48 “green” microcultures were obtained at TD, which corresponds to 0.18 ffu/culture, with a 7:1 ratio of single to multiple infections. **C** The supernatant at 30 h of one green fluorescent TD-clone was subjected to Virus-Sorting as in (A). Non fluorescent events (containing the candidate untagged rMVA EEVs) were sorted in bulk from the NEG-SW, as defined in Fig. 6A, F/T/S-treated and subjected to TD. In this particular experiment, approximately 2000 non-fluorescent events were sorted in bulk and 7/48 non-fluorescent virus infected cultures were obtained at TD, which corresponds to 0.17 ffu/culture, with a 14:1 ratio of single to multiple infections. PCR (**C**) and WB analysis (**D**) of infected cell lysates (as in Fig. 2D & E) confirmed that all four clones indeed expressed the M1-V5 transgene. Abbreviations: PC (positive control = rMVA-M1-V5-Rome-infected CEFs) (see Methods), NC1 (negative control = non-infected CEFs), NC2 (negative control = MVA-77-RED-infected CEFs)

Next, EEVs amplified from one of these green TD-clones were again subjected to Virus-Sorting, revealing a significant enrichment of green fluorescent events (Fig. 7B). To obtain untagged rMVA, we focused instead on events from the NEG-SW that we had defined above for the SHAG-EEVs (Fig. 6A, middle panel), since those EEVs likely were no longer green fluorescent as a result of having undergone Z/Z recombination-driven HAG deletion. The resulting candidate untagged rMVA-77-M1-V5 virion population was subjected to F/T/S and terminally diluted into CEF microcultures.

**Fig. 8:**
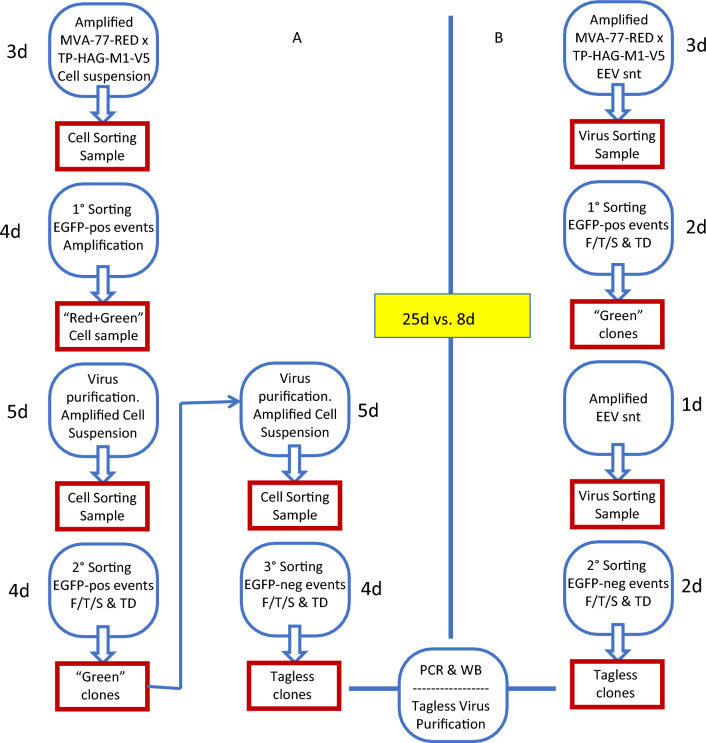
Flow-chart of untagged rMVA production. **A** Cytometry-based and **B** Virometry-based RGGSMs are set side by side. The days needed for each step are itemized. The resulting comparison (25d vs. 8d) refers to the interval between the initial Infection/Transfection step and the obtainment of the Candidate Seed Stock rMVA Clones

Four candidate clones were further analyzed by PCR and immunoblotting. Each of the four clones contained a full-length copy of the M1 gene (Fig. 7D) and displayed the V5 tag (Fig. 7E). The four clones, hence, qualify as *bona fide* untagged rMVA-77- M1-V5 candidates that can serve as Stock Seed clones for bulk production once their sequence is verified to be correct. Strikingly, the whole procedure took only about 8 days (Fig. 8B).

## Discussion

Most of the available methods for recombinant MVA production involve the selection of cells infected by recombinant constructs. Multiple rounds of infection and virus terminal dilutions are required to obtain pure clones for the desired recombinant untagged viruses. The novelty presented in this paper stems from the combination of employing a “transient” fluorescent membrane marker with the efficiency-enhancing approach of “flow virometry”, by shifting the selection step from the cells to the virions themselves. The EEV fluorescence is lost after freeze/thaw/sonication which disrupt the external membranes yielding non-fluorescent infectious virions.

We have considered the possibility to obtain untagged rMVAs directly from the first Virus-Sorting round (not shown); analysis of the virus population sorted in a NEG-SW at the first Virus-Sorting passage show that they mostly contain parental red viruses and rare untagged variants, which, analyzed by PCR, result not to contain the transgene, but to derive from parental red viruses which have lost, for trivial reasons, the tag or the flanking regions. Thus, the use of an intermediate green clone from the first Virus-Sorting passage is an essential step of the procedure, as it precludes any contamination by this unwanted population of untagged viruses.

The Virus-Sorting strategy we described here eliminates the tedium of multiple infection rounds and cell culture passages. We demonstrate that the procedure is as reliable as our previously developed RGGSM (which was already faster than most other rMVA construction methods), but much faster and less laborious. Although the benefits of flow virometry have been exploited already for many purposes, its application to efficiently select recombinant viruses is, to our knowledge, a novelty.

## Conclusions

We have streamlined the RGGSM to be more straightforward and efficient in reducing working hours and costs, because no virus purifications, and fewer cultures and sorting procedures were necessary. The virometry-based strategy for RGSSM is undoubtedly time-saving: the number of working days from the initial infection/transfection step up to obtaining untagged rMVA Stock Seed clones is reduced from 25 days required by the original cytometry-based RGGSM to 8. The streamlining of the RGGSM may thus improve on the applicability of rMVAs, for instance to produce simultaneously more candidate vaccines for various types of infectious disease and personalized vaccines for therapeutic intervention against cancer.

## Data Availability

Materials, data, and protocols described in the manuscript will be made available upon reasonable request at the corresponding author.

## Abbreviations

CEF: Chick Embryo Fibroblasts
EEV: Extracellular Enveloped Virus
RGGSM: Red-to-Green Gene SwappingMethod
EGFP: Enhanced Green Fluorescent Protein
F/T/S: Freeze, thaw, and sonication
ffu: Focus-forming units
FSC: Forward scatter
HA: Hemagglutinin
HAG: HA-EGFP fusion protein
HcRed 1.1: Fluorescent red marker
HRP: Horse Raddish Peroxidase
IMV: Intracellular Mature Virus
m.o.i.: Multiplicity of infection
MVA-77-RED: Acceptor virus carrying HcRed 1.1
MVA: Modified Virus Ankara
p.i.: Post infection
P7.5: Vaccinia early/late promoter
rMVA: Recombinant MVA
SHAG: “Stable green” HAG construct
sP: Vaccinia Synthetic Promoter
SSC: Side scatter
TD-clones: Clones derived from TD
TD: Terminal Dilution
TP-G: Transfer Plasmid carryng EGFP
TP-HAG: Transfer Plasmid carryng HAG
TP-SHAG: Transfer Plasmid carryng SHAG
TP: Transfer Plasmid
WB: Western blot
Z-repeats: 283-Bp sequences from E.coli LacZ gene
