# Correction: Time- and cost-effective production of untagged recombinant MVA by flow virometry and direct virus sorting

**DOI:** 10.1186/s12967-023-04480-1

**Published:** 2023-09-28

**Authors:** Daniela Boselli, Maddalena Panigada, Simona Di Terlizzi, Monica Romanò, Emanuele Canonico, Chiara Villa, Claudia Minici, Eelco van Anken, Elisa Soprana, Antonio G. Siccardi

**Affiliations:** 1 Division of Genetics and Cell Biology, San Raffaele Scientific Institute, Milan, Italy; 2 FRACTAL-San Raffaele Scientific Institute, Milan, Italy; 3 Università VitaSalute San Rafaele, Milan, Italy

**Correction: Journal of Translational Medicine (2023) 21:495** **https://doi.org/10.1186/s12967-023-04353-7**

Following publication of the original article [1], we have been notified that the author name of Daniela Boselli was mentioned incorrectly. The correct name was updated in this erratum.
